# Apoptosis, mastocytosis, and diminished adipocytokine gene expression accompany reduced epididymal fat mass in long-standing diet-induced obese mice

**DOI:** 10.1186/1476-511X-10-198

**Published:** 2011-11-03

**Authors:** Mehmet M Altintas, Maria A Rossetti, Behzad Nayer, Alvaro Puig, Patricia Zagallo, Luis M Ortega, Kevin B Johnson, George McNamara, Jochen Reiser, Armando J Mendez, Ali Nayer

**Affiliations:** 1 Department of Medicine, Miller School of Medicine, University of Miami, Miami, FL, USA; 2 Diabetes Research Institute and Division of Endocrinology, Diabetes and Metabolism, University of Miami, Miami, FL, USA; 3 College of Engineering, University of Miami, Coral Gables, FL, USA; 4 Analytical Imaging Core Facility, Diabetes Research Institute, and UM/Sylvester Comprehensive Cancer Center, University of Miami, Miami, FL, USA

**Keywords:** Obesity, adipose tissue, inflammation, mast cells, macrophages, crown-like structures, apoptosis, adipokines

## Abstract

**Background:**

Obesity is characterized by increased cell death and inflammatory reactions in the adipose tissue. Here, we explored pathophysiological alterations taking place in the adipose tissue in long-standing obesity. In the epididymal fat of C57BL/6 mice fed a high-fat diet for 20 weeks, the prevalence and distribution of dead adipocytes (crown-like structures), mast cells (toluidine blue, mMCP6), macrophages (F4/80), and apoptotic cells (cleaved caspase-3) were measured. Moreover, gene and/or protein expression of several adipocytokines (leptin, adiponectin, TNF-α, IL-10, IL-6, MCP-1), F4/80, mMCP6, cleaved caspase-3 were determined.

**Results:**

We observed that the epididymal fat mass was lower in obese than in lean mice. In obese mice, the epididymal fat mass correlated inversely with body weight and liver mass. Dead adipocytes, mast cells, macrophages, and apoptotic cells were abundant in the epididymal fat of obese mice, especially in the rostral vs. caudal zone. Accordingly, mMCP6, F4/80, and cleaved caspase-3 gene and/or protein expression was increased. Conversely, adiponectin, leptin, IL-6, and MCP-1 gene expression levels were lower in the epididymal fat of obese than lean mice. Although TNF-α and IL-10 gene expression was higher in the epididymal fat of obese mice, their expression relative to F4/80 and mMCP6 expression were lower in the heavily infiltrated rostral than caudal zone.

**Conclusions:**

This study demonstrates that in mice with long-standing obesity diminished gene expression of several adipocytokines accompany apoptosis and reduced mass of the epididymal fat. Our findings suggest that this is due to both increased prevalence of dead adipocytes and altered immune cell activity. Differential distribution of metabolically challenged adipocytes is indicative of the presence of biologically diverse zones within the epididymal fat.

## Background

A steady increase in the prevalence of obesity has been observed worldwide. Increased caloric intake and decreased physical activity are major contributors. Obesity is often accompanied by activation of inflammatory signaling pathways and alterations in systemic and local levels of inflammatory cytokines [1]. Many of these cytokines, including tumor necrosis factor- α (TNF-α), interleukin-6 (IL-6), interleukin-10 (IL-10), and monocyte chemotactive protein-1 (MCP-1) have profound effects on metabolism [1]. The adipose tissue is directly involved in the chronic inflammatory state that is observed in obesity. Cells of innate immune system, such as macrophages [2], mast cells [3,4], and neutrophils [5] accumulate in the adipose tissue in obesity. Moreover, cells of adaptive immunity, including regulatory T cells [6,7], CD8^+ ^T cells [7], and natural killer T cells [8] are also involved in adipose tissue inflammation in obesity.

Although the details of the underlying mechanisms that provoke inflammation in the adipose tissue in obesity remain largely elusive, adipocyte injury and death play a central role [9]. Based on ultrastructural and immunohistochemical alterations, it was proposed that many adipocytes in white adipose tissue of obese rodents and humans demonstrate characteristic features of necrosis, but not apoptosis [9]. A recent study, however, showed that both mitochondrial- and death receptor-mediated caspase activation and adipocyte apoptosis were increased in the adipose tissue of obese humans and diet-induced obese mice [10]. Regardless of the pathway(s) involved, metabolically challenged adipocytes are more prevalent and accompanying inflammatory reactions are more severe in visceral than in subcutaneous fat in obesity [3,11]. There is also a remarkable heterogeneity in the severity of adipose tissue inflammation in different visceral fat depots in obese subjects [3,12].

Although much emphasis has been placed on the understanding of molecular, biochemical, and immunological mechanisms involved in adipose tissue inflammation in obesity, little is known about immune responses taking place in the adipose tissue during the advanced stages of obesity. In the present study, we examined the epididymal fat of mice with long-standing obesity and determined: 1) the density and distribution of dead adipocytes, macrophages, and mast cells throughout the fat depot, 2) gene expression levels of several adipocytokines, 3) the prevalence of apoptosis, and 4) the relation among apoptosis, adipocytokine gene expression, and the degree of inflammatory cell infiltration. We showed that reduced mass of the epididymal fat in mice with long-standing obesity is accompanied by divergent distribution of crown-like structures, apoptotic cells, mast cells, and macrophages leading to diminished adipocytokine gene expression.

## Methods

### Experimental animals

We followed the 'Principles of laboratory animal care' established by the National Institutes of Health. The Institutional Animal Care and Use Committee of the University of Miami specifically approved this study (IACUC protocol number 08-245). Blood samples and tissues were from a recent study [3]. Male C57BL/6 mice were purchased from Taconic (Hudson, NY) and acclimated for two weeks before the beginning of the study. Mice were randomized to either a high-fat diet (HFD) consisting of 60% calories from fat (D12492) or a low-fat diet (LFD) consisting of 10% calories from fat (D12450B) at the age of 6 weeks and were sacrificed when 6 months old (Research Diets Inc., St. Louis, MO). Mice were weighed with a Scout Pro balance SP202 (Ohaus, Pine Brook, NJ). Organ weights were measured with a Sartorius ED124S Analytical Balance (Sartorius, Bohemia, NY).

### Glucose, insulin and cholesterol assays

After overnight fasting (15 hours), blood was sampled from the tail of unanesthetized mice. Blood glucose concentrations were measured using a Contour glucometer (Bayer, Tarrytown, NY). Serum insulin concentrations were measured by immunoassay following manufacturer's instructions (Crystal Chem, Downer Grover, IL). Serum cholesterol concentrations were determined by Cholesterol LiquiColor enzymatic assay (Stanbio Laboratory, Boerne, TX). Homeostasis Model of Assessment - Insulin Resistance (HOMA-IR) was calculated using the formula: fasting glucose (mg/dl) × fasting insulin (mU/L)/405.

### The anatomy of the epididymal fat in the mouse

**Figure 1:**
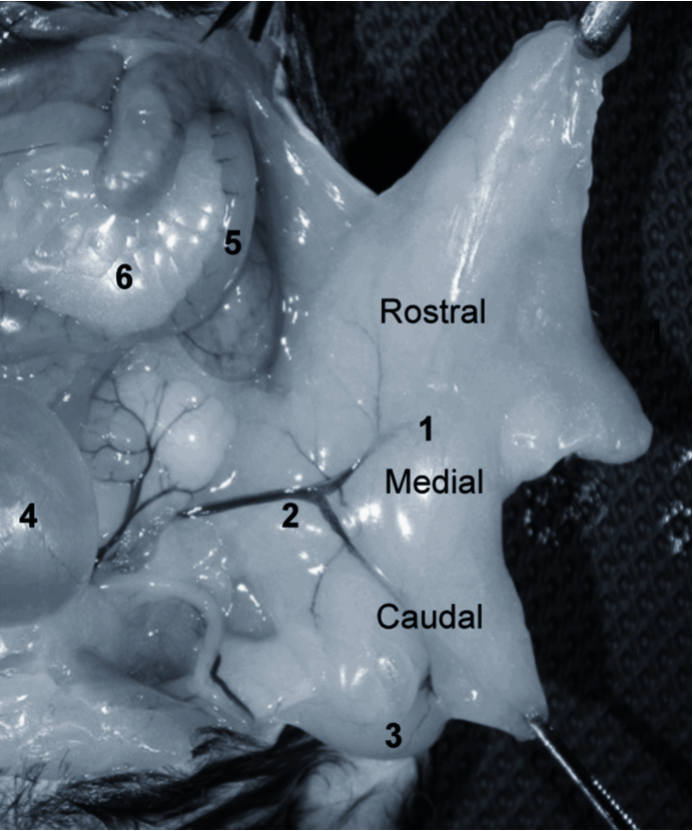
**The epididymal fat in the male mouse**. To demonstrate abdominal organs, the anterior abdominal wall was removed and the left epididymal fat (1) was deflected to the left. The epididymal fat was divided into a caudal, medial, and rostral zone relative to the location of spermatic blood vessels (2). The left testis (3), the urinary bladder (4), a small bowel loop (5), and attached mesenteric fat (6) are also shown.

The epididymal fat is an anatomically distinct and metabolically active abdominal fat depot widely used to study adipose tissue biology in rodents [2-12]. The paired epididymal fat is attached caudally to the ipsilateral testis and epididymis and extends proximally toward the diaphragm (Figure 1). The epididymal fat incorporates spermatic blood vessels and accompanies them medially to the retroperitoneum [13]. Using spermatic blood vessels as a landmark, the epididymal fat can be divided into three zones: 1) medial zone, located around the spermatic artery and its main branches, 2) caudal zone, attached to the testis and epididymis, and 3) rostral zone, the loose end proximal to the medial zone. We quantified the density of mast cells and crown-like structures (CLS) in all three zones. A crown-like structure consists of several macrophages enveloping an adipocyte [9]. Protein and gene expression was measured only in the caudal and rostral zones that showed the greatest difference in the density of mast cells and CLS (see below).

### Histology

Tissues were fixed in Carnoy's fixative and embedded in paraffin. Five micron-thick sections were cut, baked overnight at 60°C, deparaffinized in xylene, rehydrated in a graded ethanol series, and then stained. To demonstrate mast cells, toluidine blue staining was carried out by briefly submerging tissue sections in 0.1% aqueous toluidine blue (EMS, Hatfield, PA) [3]. In addition, the esterase activity of mast cells was detected using naphthol AS-D chloroacetate as substrate as previously described [3].

### Immunohistochemical staining for macrophage F4/80

As previously described, following quenching endogenous peroxidase activity and heat-induced epitope retrieval, deparaffinized tissue sections were blocked with rabbit serum and sequentially incubated with a monoclonal antibody against macrophage F4/80 (1:100; AbD Serotec, Raleigh, NC) or isotype control, an appropriated secondary antibody, avidin-biotin complex, and 3,3'-diaminobenzidine [3].

### Quantitation of mast cells and CLS

Toluidine blue-stained sections were used to count mast cells. F4/80-stained sections were used to count CLS. Mast cells and CLS were counted in 20 high-power fields (400X) in the caudal, medical, and rostral zones of the epididymal fat. Density was expressed as mast cells or CLS per mm^2 ^of tissue section. Light microscopic images were acquired using a Leica DMLB microscope with a Leica DFC420 C color camera (Leica, Bannockburn, IL).

### Immunofluorescence staining for cleaved caspase-3

Antigen retrieval was carried out in 10 mM citrate buffer, pH 6.0, heated to 95-100°C for 15 minutes in a microwave. Tissue sections were blocked with 5% goat serum for 30 minutes and then sequentially incubated with rabbit anti-mouse cleaved caspase-3 antibody for 60 minutes (1:200; Cell Signaling, Danvers, MA), Alexa Fluor 594 goat anti-rabbit IgG for 60 minutes (1:1,000; Invitrogen, Carlsbad, CA), and DAPI (4',6-diamidino-2-phenylindole) for 5 minutes (0.1 μg/ml, Invitrogen, Carlsbad, CA). All incubations took place in a humid chamber at room temperature. Sections were rinsed with PBS between incubations.

### Electron microscopy

After immersion fixation in 0.1 M phosphate buffer containing 2% glutaraldehyde and 2% paraformaldehyde, pH 7.4, adipose tissue samples were post-fixed in 0.1 M phosphate buffer containing 1% osmium tetroxide for 2 hours at 4°C. Samples were then dehydrated in a graded acetone series, infiltrated and embedded in Spurr's epoxy resin. Tissue sections were cut at 50 nm (Reichert Ultracut S microtome), retrieved onto 300 mesh copper grids, and contrasted with uranyl acetate and lead citrate. Sections were examined using a Morgagni 268 transmission electron microscope (FEI, Hillsboro, OR) and images were acquired with an AMT Advantage 542 CCD camera system (Advanced Microscopy Techniques, Danvers, MA).

### Protein extraction and immunoblotting

100 mg adipose tissue was homogenized in 0.5 ml ice-cold lysis buffer containing 50 mM Tris-HCl (pH 7.4), 150 mM NaCl, 1% NP-40, 0.5% sodium deoxycholate, 0.1% SDS, 5 mM EDTA, 1 mM EGTA (Boston BioProducts, Ashland, MA) supplemented with a cocktail of protease inhibitors (Complete Mini, Roche Applied Science, Indianapolis, IN). Homogenates were agitated on a rotator for 1 hour at 4°C followed by centrifugation at 13,000 rpm for 20 minutes at 4°C. The middle phase of lysate was subjected to polyacrylamide gel electrophoresis (NuPAGE Bis-Tris system, Invitrogen, Carlsbad, CA). The following antibodies were used for immunoblotting: cleaved caspase-3 (1:1,000; Cell Signaling, Danvers, MA), mMCP6 (1:1,000; R&D Systems, Minneapolis, MN), and GAPDH (1:10,000; Abcam, Cambridge, MA). Signal intensities were measured using ImageJ (NIH, Bethesda, MD) and target protein-to-GAPDH ratio was used for statistical analysis.

### Quantitative real-time PCR

Total RNA was isolated from 50 mg of adipose tissue using the RNeasy kit (Qiagen, Valencia, CA) and treated with DNAse I (Applied Biosystems, Carlsbad, CA). cDNA was synthesized using the reverse transcriptase reaction (High-Capacity cDNA Reverse Transcription Kits, Applied Biosystems) following manufacturer's protocol. Primers for polymerase chain reactions (PCR) were from Applied Biosystems; adiponectin (catalogue # Mm00456425_m1), leptin (Mm00434759_m1), TNF-α (Mm00443258_m1), IL-10 (Mm00439614_m1), IL-6 (Mm00446190_m1), MCP-1 (Mm00441242_m1), mMCP6 (Mm01301240_g1), F4/80 (Mm00802529_m1), and 18S (Hs03928990_G1). Quantitative gene expression by real-time PCR was performed using 40 ng of cDNA amplified with TaqMan Universal PCR Master Mix (#4352042) and reactions run using universal cycling conditions on a StepOnePlus real-time PCR System (Applied Biosystems). Samples were analyzed in triplicate and were normalized to the housekeeping gene, 18S. To analyze relative quanitation (RQ), the ΔΔCT (threshold cycle) method was used [14]. The RQ results were expressed as the fold change in gene expression relative to expression levels in the caudal epididymal fat of lean mice. *Statistics *Results are presented as mean ± SEM. Unpaired Student's t test was used to assess for statistically significant differences between groups. Comparisons between multiple groups were carried out using one-way analysis of variance (ANOVA) with Tukey post-hoc analysis. GraphPad Prism software (5.0a) was used for calculations (GraphPad Software, La Jolla, CA).

## Results

### Mice with long-standing diet-induced obesity had a lower epididymal fat mass

Compared with mice fed on a LFD (n = 10), HFD-fed mice (n = 10) demonstrated significantly higher fasting blood glucose, serum insulin and cholesterol concentrations, and HOMA-IR (Table 1). In addition, HFD-fed mice had significantly higher body weights and larger livers and inguinal subcutaneous fat depots (Table 1). However, the epididymal fat of diet-induced obese mice weighed less than that of lean controls (Table 1).

**Table 1:** Metabolic characteristics of mice

Variables	obese (n = 10)	lean (n = 10)	*p *value
Body weight (g)	48.7 ± 1.0	36.7 ± 0.7	< 0.001

Epididymal fat weight (g)	0.56 ± 0.02	0.86 ± 0.04	< 0.001

Inguinal fat weight (g)	1.33 ± 0.05	0.60 ± 0.03	< 0.001

Liver weight (g)	3.05 ± 0.19	1.81 ± 0.06	< 0.001

Blood glucose (mmol/l)	8.44 ± 0.72	4.27 ± 0.33	< 0.001

Serum insulin (ng/ml)	2.3 ± 0.2	0.6 ± 0.1	< 0.001

HOMA-IR	25.3 ± 3.7	3.4 ± 0.6	< 0.001

Serum cholesterol (mmol/l)	3.99 ± 0.21	3.31 ± 0.16	< 0.001

### In obese mice, epididymal fat mass correlated inversely with liver mass and body weight

**Figure 2:**
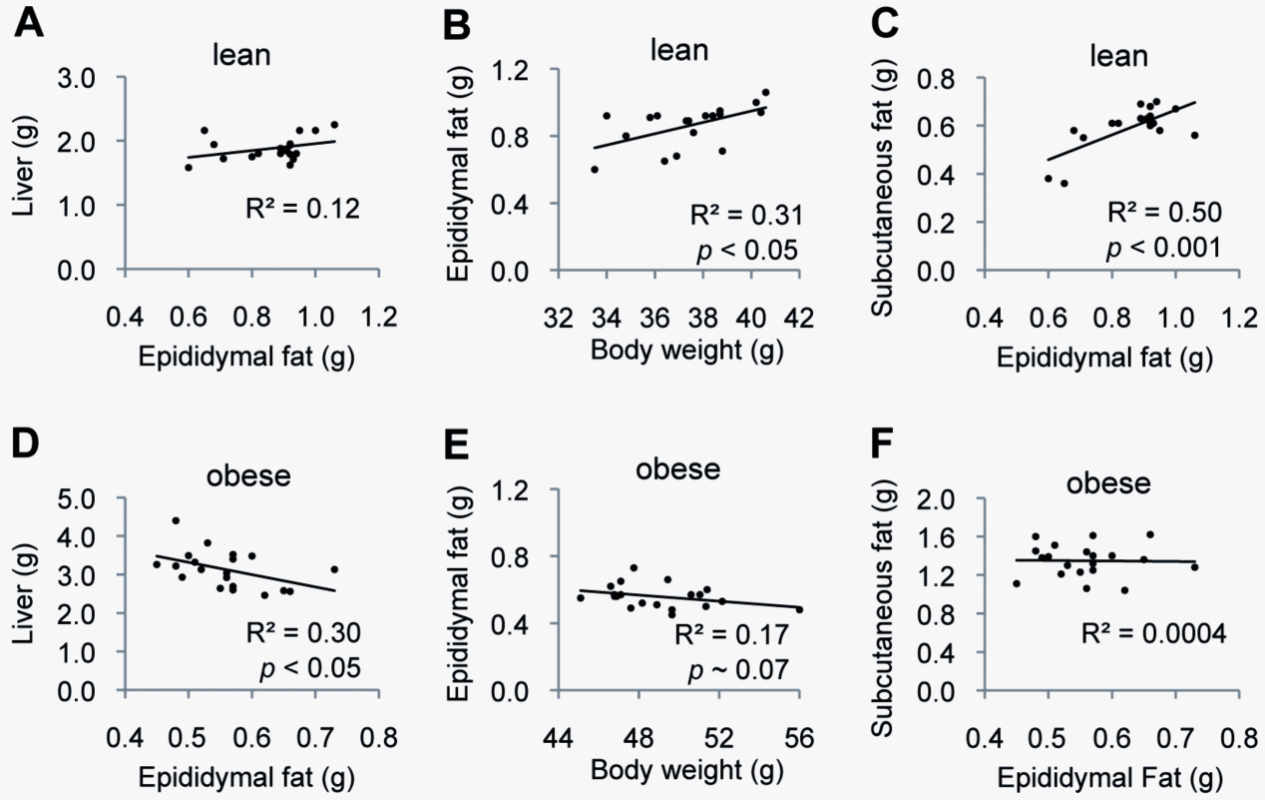
**The body weight and the weights of the epididymal and inguinal subcutaneous fat depots**. Relations between the body weight and the weights of the epididymal and inguinal fat depots in lean (A-C) and longstanding obese (D-F) mice are shown. The weight of the epididymal fat correlated inversely with the weight of the liver and the body weight in long-standing obese mice.

In lean mice, there was a trend for positive correlation between the mass of the epididymal fat and the mass of the liver (Figure 2A). Moreover, the mass of the epididymal fat correlated positively with body weight and the mass of the inguinal subcutaneous fat (Figure 2B-C). In obese mice, however, the mass of the epididymal fat correlated inversely with the mass of the liver (Figure 2D). Moreover, there was a trend for an inverse correlation between the mass of the epididymal fat and the body weight (Figure 2E). There was no correlation between the mass of the epididymal fat and that of the inguinal subcutaneous fat (Figure 2F).

### Mast cells were distributed differentially in the epididymal fat

**Figure 3:**
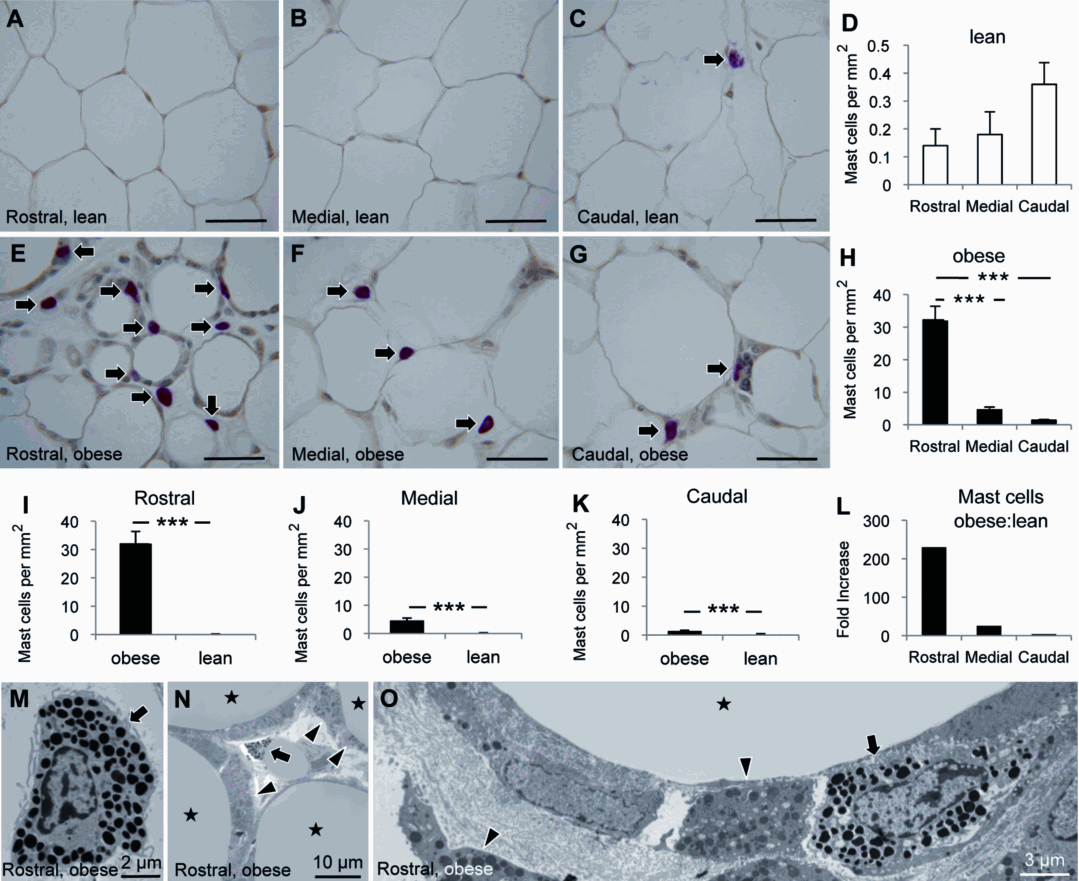
**Differential distribution of mast cells in the epididymal fat**. The distribution of mast cells in the epididymal fat from lean (A-D) and diet-induced obese (E-H) mice (n = 10 per group) are shown. The density of mast cells in the rostral, medial, and caudal epididymal fat was compared between lean and obese mice (I-L). Ultrastructural examination revealed frequent mast cells (arrows) intermingled with macrophages (arrowheads) in the expanded interstitial space between adipocytes (stars) in obese mice (M-O). Scale bars: 50 μm or as indicated. *** *p *< 0.001.

In lean mice, there was a trend for higher mast cell density in caudal (0.4 ± 0.1 cells per mm^2^) than in medial (0.2 ± 0.1 cells per mm^2^) and rostral (0.1 ± 0.1 cells per mm^2^) zones (Figures 3A-D). In obese mice, however, the density of mast cells increased substantially and progressively from caudal (1.4 ± 0.2 cells per mm^2^) to rostral (32.2 ± 4.4 cells per mm^2^) (Figures 3E-H). Although obesity was accompanied by a significant increase in mast cells in all three zones (Figures 3I-K), the greatest increase was observed in the rostral epididymal fat where a 230-fold increase was noted (Figure 3L). Ultrastructural examination confirmed the presence of frequent mast cells in the rostral epididymal fat of obese mice (Figures 3M-O). Mast cells were intermingled with macrophages in the expanded interstitial space between adipocytes (Figures 3N,O).

### Mouse mast cell protease-6 (mMCP6) protein expression was increased in the epididymal fat of obese mice

**Figure 4:**
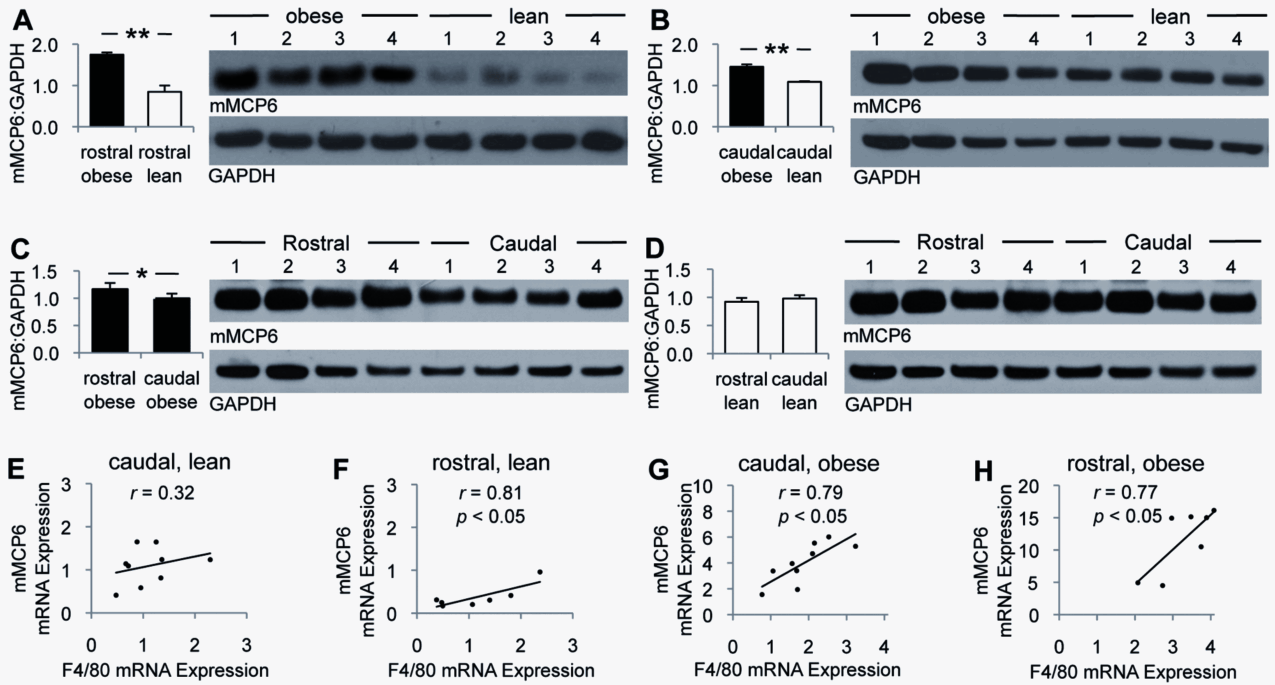
**Mouse mast protease-6 (mMCP6) expression in the epididymal fat**. Western blot analysis of mMCP6 protein expression in the rostral and caudal epididymal fat from lean (n = 4, white bars) and diet-induced obese (n = 4, black bars) mice is shown (A-D). Correlations between mMCP6 and macrophage-specific F4/80 gene expression are also demonstrated (E-H). * *p *< 0.05, ** *p *< 0.01.

Western blot analysis demonstrated that mMCP6 expression was higher in the epididymal fat of obese vs. lean mice (Figures 4A,B). Although mMCP6 protein expression was higher in rostral than in caudal epididymal fat of obese mice, the difference was not significant in lean mice (Figures 4C,D). In addition, there was a positive correlation between mMCP6 and F4/80 gene expression in the epididymal fat, especially in obese mice (Figures 4E-H).

### CLS were distributed differentially in the epididymal fat

**Figure 5:**
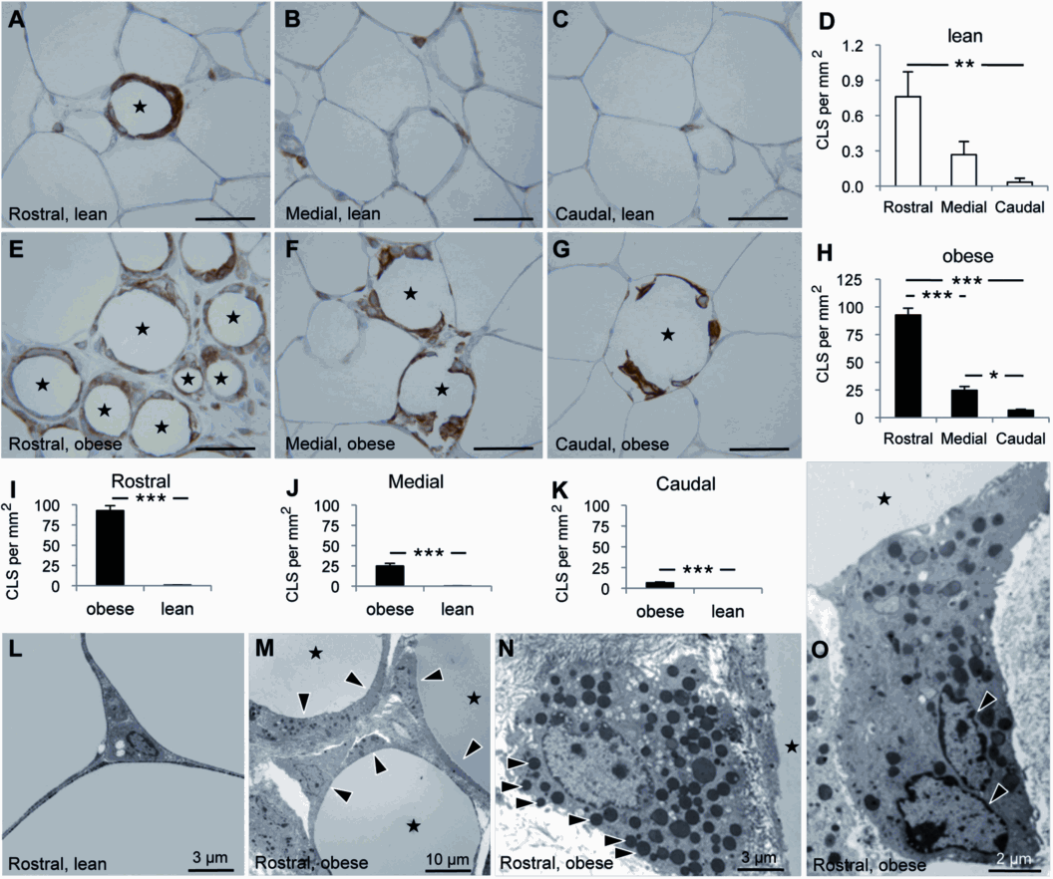
**Differential distribution of crown-like structures (CLS) in the epididymal fat**. The densities of CLS in the epididymal fat from lean (A-D) and diet-induced obese (E-H) mice (n = 10 per group) are shown. The density of CLS in the rostral, medial, and caudal epididymal fat was compared between lean and obese mice (I-K). Ultrastructural examination demonstrated a delicate interstitium in the epididymal fat from lean mice (L). In obese mice, the expanded interstitial space contained macrophages (arrowheads), many of which enveloped scattered adipocytes forming CLS (stars) (M). While many macrophages were packed with lipid droplets (N), some were multinucleated (O). Scale bars: 50 μm or as indicated. * *p *< 0.05, ** *p *< 0.01, *** *p *< 0.001.

In lean mice, CLS were more prevalent in rostral (0.8 ± 0.2 CLS per mm^2^) than in medial (0.3 ± 0.1 CLS per mm^2^) and caudal (0.03 ± 0.03 CLS per mm^2^) epididymal fat (Figures 5A-D). Similarly, the density of CLS was higher in rostral (92.6 ± 6.2 CLS per mm^2^) than in medial (24.7 ± 3.4 CLS per mm^2^) and caudal (6.7 ± 0.8 CLS per mm^2^) epididymal fat of obese mice (Figures 5E-H). Obesity was accompanied by a substantial increase in CLS in all three zones (Figures 5I-K). Ultrastructural examination of the epididymal fat of lean mice demonstrated a delicate interstitium separating adipocytes (Figure 5L). In obese mice, however, macrophages and mast cells accumulated in the expanded interstitium and often enveloped adipocytes forming CLS (Figure 5M). Furthermore, many macrophages contained numerous lipid droplets (Figure 5N), while some were multinucleated (Figure 5O).

### Reduced leptin, adiponectin, IL-6, and MCP-1 gene expression accompanied apoptosis in the epididymal fat of obese mice

**Figure 6:**
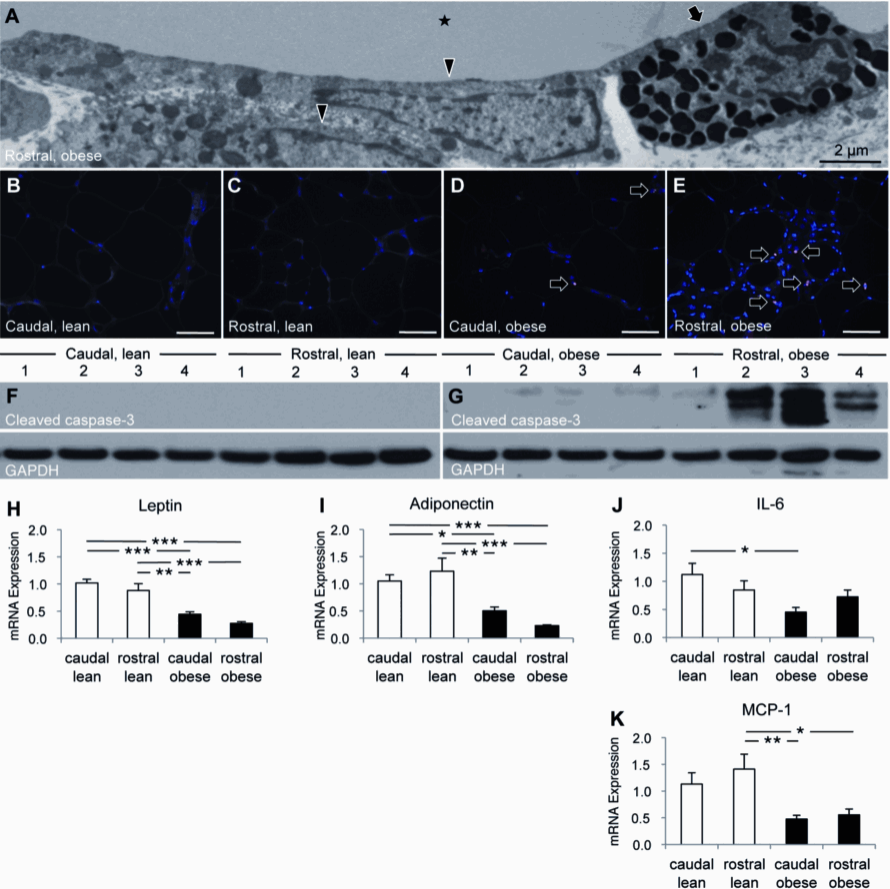
**Adipocytokine gene expression and apoptosis in the epididymal fat**. Ultrastructural examination of the epididymal fat from diet-induced obese mice demonstrated frequent macrophages (arrowheads) and mast cells (arrow) abutting lipid cores (star) with no evidence of the cytoplasm of adipocytes (A). The expression of cleaved caspase-3 was determined by immunostaining (arrows, B-E) and Western blot analysis (F-G). Leptin, adiponectin, IL-6, and MCP-1 gene expression was determined in the caudal and rostral epididymal fat from lean (white bars) and obese (black bars) mice (H-K). Scale bars: 50 μm or as indicated. * *p *< 0.05, ** *p *< 0.01, *** *p *< 0.001.

On ultrastructural examination of the epididymal fat of obese mice, frequent macrophages and mast cells were found abutting lipid cores with no evidence of adipocyte cytoplasm indicating loss of adipocytes (Figure 6A). Rarely observed in the epididymal fat of lean mice, apoptosis, detected by immunostaining for cleaved caspase-3, was frequent in the epididymal fat of obese mice, especially in the rostral zone (Figures 6B-E). Western blot analysis confirmed increased cleaved caspase-3 protein expression in the rostral, and to a lesser degree in the caudal, epididymal fat of obese mice (Figures 6F,G). Compared to lean controls, leptin, adiponectin, IL-6, and MCP-1 gene expression was reduced in the epididymal fat of obese mice (Figures 6H-K). There was also a trend for lower leptin and adiponectin expression in the rostral than in the caudal epididymal fat of obese mice (Figures 6H, I).

### Discordance between TNF-α and IL-10 gene expression and immune cell accumulation in the epididymal fat of obese mice

**Figure 7:**
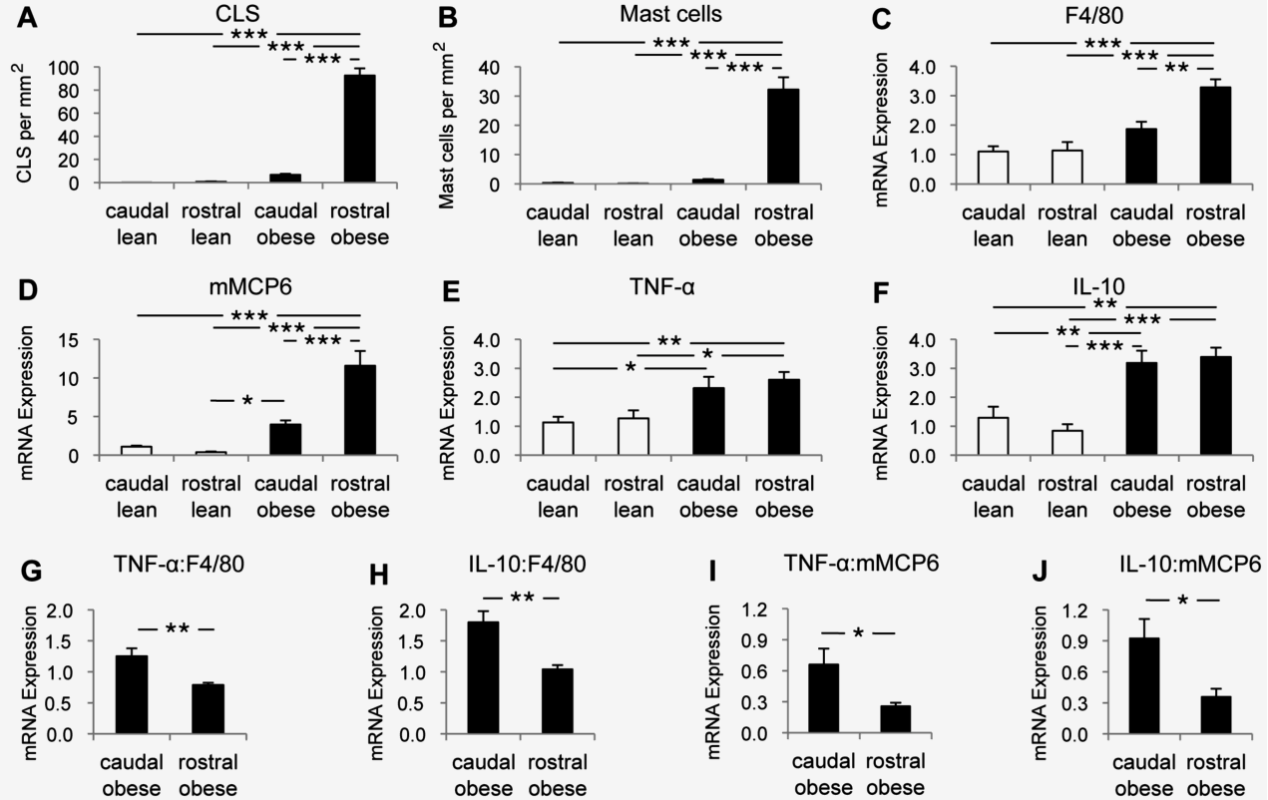
**TNF-α and IL-10 gene expression in the epididymal fat**. The distribution of CLS and mast cells and the expression of F4/80, mMCP6, TNF-α and IL-10 genes in the caudal and rostral epididymal fat from lean (white bars) and diet-induced obese (black bars) mice are shown (A-F). TNF-α and IL-10 gene expression relative to F4/80 and mMCP6 gene expression is also shown (G-J) * *p *< 0.05, ** *p *< 0.01, *** *p *< 0.001.

In the epididymal fat of obese mice, the densities of mast cells and CLS were 22- and 14-fold, respectively, higher in the rostral vs. caudal zone (Figure 7A,B). Similarly, F4/80 and mMCP6 gene expression levels were higher in the rostral vs. caudal zone (Figures 7C,D). Although obesity was accompanied by increased TNF-α and IL-10 gene expression levels in the epididymal fat, there was no significant difference between the caudal and the heavily infiltrated rostral zones (Figures 7E,F). In fact, expression levels of TNF-α and IL-10 genes relative to those of F4/80 and mMCP6 genes were significantly lower in the heavily infiltrated rostral vs. caudal epididymal fat (Figures 7G-J).

### In the heavily infiltrated rostral epididymal fat of obese mice, positive correlations among TNF-α, IL-6, MCP-1, and IL-10 gene expression levels were lost

**Figure 8:**
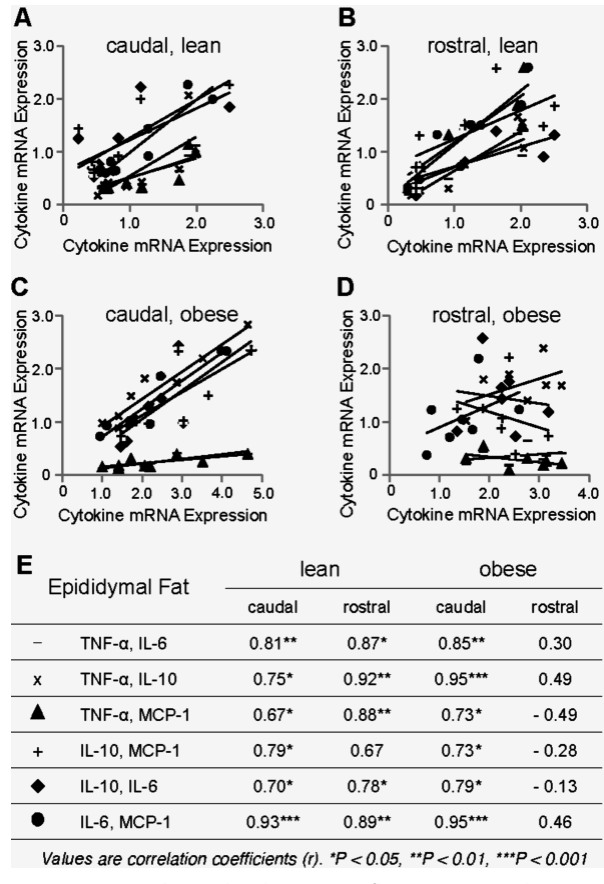
**Interrelationship between inflammatory cytokines in the epididymal fat**. The correlations among expression levels of TNF-α, IL-10, IL-6, and MCP-1 genes in the caudal and rostral epididymal fat from lean and diet-induced obese mice are shown.

Positive correlations were noted among expression levels of TNF-α, IL-6, MCP-1, and IL-10 genes in the epididymal fat of lean mice (Figures 8A,B). In obese mice, positive correlations persisted in the caudal epididymal fat (Figure 8C). However, in the heavily infiltrated rostral epididymal fat of obese mice, there were no statistically significant correlations between gene expression levels of these cytokines (Figures 8D,E).

## Discussion

While immune responses in the adipose tissue of mice fed on a high-fat diet over a relatively short period of time are studied extensively, little is known about advanced stages of adipose tissue inflammation in long-standing obesity. Here, we examined the effects of long-term high-fat feeding on adipocytokine gene expression in the epididymal fat of mice with long-standing obesity and correlated the findings with the extent of apoptosis and inflammatory cell infiltration. We showed that extensive loss of adipocytes in this fat depot was associated with reduced fat mass and diminished leptin, adiponectin, IL-6, and MCP-1 gene expression levels. We also showed that cell death and inflammatory cell infiltration occurred predominantly in the rostral zone of the epididymal fat of obese mice. In addition, we found an increase in the expression levels of TNF-α and IL-10 genes in the epididymal fat of obese mice. However, TNF-α and IL-10 expression levels relative to those of F4/80 and mMCP-6 was significantly lower in the heavily infiltrated rostral than in caudal epididymal fat.

There are structural and functional differences between the adipose tissue found in different anatomical locations. For example, subcutaneous and visceral fat differ in immune cell composition, insulin sensitivity, lipolysis, expression of glucose transporters, and obesity-associated adipocyte death and attendant inflammation [3,15-17]. Biological and immunological differences also exist among various visceral fat depots. For example, we have shown that the prevalence of both mast cells and macrophage CLS are higher in epididymal and perinephric fat than in mesenteric fat of diet-induced obese mice [3]. Emerging evidence indicates that there is also substantial heterogeneity in the structure and function of superficial and deep abdominal subcutaneous fat [18]. Here, we show for the first time that there are distinct zones (regions) within an abdominal fat depot that exhibit different level of susceptibility to metabolic challenges. Although the mechanisms underlying adipocyte injury and death in obesity are under investigation, it has been proposed that exaggerated metabolic demands trigger stress responses in adipocytes that could lead to cell injury and death [19]. In addition, the disequilibrium between oxygen demand and supply in expanding adipose tissues in obesity can lead to local hypoxia, which might have a role in the pathogenesis of adipocyte dysfunction, altered adipokine expression, and adipose tissue inflammation [20]. Based on these observations, it could be argued that, within an abdominal fat depot, there are functionally diverse populations of adipocytes that show different levels of tolerance to metabolic challenges. Alternatively, adipose tissue expansion in obesity might not be uniform throughout the same depot leading to lower oxygen tension in areas of fat depots undergoing a more rapid expansion. Considering the severity of pathological changes in the present study, we elected not to determine the degree of hypoxia in the caudal and rostral epididymal fat of obese mice.

Consistent with a previous report, the loss of the epididymal fat mass in obese mice was inversely related to the weight of the liver [11]. Therefore, it could be argued that lipids released from dead adipocytes, if not locally metabolized by phagocytes, would possibly find their way to the liver and/or other fat depots. Alternatively, the diminished ability of a smaller epididymal fat depot containing many dysfunctional or dead adipocytes would result in energy storage in the liver and/or other fat depots.

Inflammatory cytokines play a significant role in the pathogenesis of insulin resistance [1]. IL-6 is reported to induce insulin resistance in the liver, the skeletal muscle, and 3T3-L1 adipocytes [21-23]. While IL-6 secretion from explanted adipose tissue from obese human subjects is well documented [24], studies comparing IL-6 protein and/or gene expression between lean and obese subjects are rather sparse. Nevertheless, a recent study showed that IL-6 gene expression was increased in the epididymal fat of mice fed on a high-fat diet (42% calories from fat) for 6 weeks [10]. Similarly, the perigonadal adipose tissue of 8-week old obese KK-*A*^*y *^mice, a model of genetic obesity, demonstrated elevated IL-6 gene expression levels [25]. Loss-of function and gain-of-function mutations showed that MCP-1 play a role in macrophage accumulation and insulin sensitivity in the adipose tissue [26]. MCP-1 was overexpressed in white adipose tissue of mice fed on a high-fat diet (56% calories from fat) for 12 weeks [26]. In addition, compared to mice on control diet, MCP-1 gene expression was greater in the epididymal fat of 20-week old mice after 16 weeks of high fat feeding (45% calories from fat) [2]. In contrast to these studies, here we used mice fed on a diet with higher fat content (60% calories from fat) for a longer period of time (20 weeks) and showed a reduction in IL-6 and MCP-1 gene expression in the epididymal fat. Strissel et al. randomized 5-week-old C57BL/6 mice to either a low-fat diet (10% calories from fat) or a high-fat diet (60% calories from fat) for up to 20 weeks and compared adipocytokine gene expression in the epididymal fat of obese mice of different age [11]. They observed a trend for decreased MCP-1 and IL-6 gene expression between weeks 16 and 20 of high-fat feeding [11]. Since IL-6 and MCP-1 are secreted mainly by the stromal vascular fraction of the adipose tissue [27], we argue that reduced IL-6 and MCP-1 gene expression in the epididymal fat of mice with long-standing obesity is a manifestation of reduced pro-inflammatory activity of immune cells in the adipose tissue.

A potent anti-inflammatory cytokine, IL-10 is secreted by macrophages, dendritic cells, mast cells, and subsets of B- and T-lymphocytes [28]. Recent evidence suggests that IL-10 has favorable effects on insulin sensitivity and adipocyte function. Hyperinsulinemic-euglycemic clamp studies showed that IL-10 prevents IL-6-induced insulin resistance in the liver and the skeletal muscle in mice [21]. In addition, IL-10 decreased MCP-1 production and ameliorated TNF-α-induced inhibition of insulin-stimulated glucose uptake by 3T3-L1 cells [29]. Consistent with previous reports, we found increased IL-10 and TNF-α gene expression in the epididymal fat of obese mice [30,31]. However, IL-10 and TNF-α gene expression relative to those of F4/80 and mMCP6 was reduced in the rostral epididymal fat where macrophages and mast cells were abundant. Since IL-10 and TNF-α are expressed and released mainly from the stromal vascular fraction of the adipose tissue [27], these findings provide further evidence for altered inflammatory activity of immune cells in the epididymal fat depot of mice with long-standing obesity.

Adiponectin and leptin are cytokines produced mainly by adipocytes. Adiponectin promotes insulin sensitivity and, by modulating innate and adaptive immune responses, exerts potent anti-inflammatory effects [32]. Consistent with previous reports, here we found reduced adiponectin gene expression in the epididymal fat of obese mice [33,34]. However, our finding of reduced leptin gene expression in the epididymal fat of mice with long-standing obesity is not consistent with previous reports [20,35]. We argue that high numbers of dysfunctional or dead adipocytes in a fat depot could lead to the reduced expression of proteins that are primarily synthesized by adipocytes. In fact, the loss of adipocytes, at least in part by apoptosis, in the epididymal fat of obese mice in the present study was so severe that it led to a decrease in fat mass, a finding that has also been observed by others [11]. Consistent with these findings, we recently reported reduced adiponectin and leptin gene expression in the visceral and inguinal subcutaneous fat of aP2-nSREBP-1c transgenic mice, a model of lipodystrophy [36]. Since mice have several distinct fat depots in various anatomical locations, the contribution of diminished leptin and adiponectin gene expression in the epididymal fat to their circulating concentrations in obese mice remains to be determined. However, considering paracrine actions of leptin and adiponectin, it could be argued that decreased leptin and adiponectin expression would affect the behavior of adipocytes and cells of the stromal vascular fraction, including immune cells, present in the adipose tissue. This is of particular interest considering the differences in relative TNF-α and IL-10 expression between the caudal and the rostral epididymal fat of obese mice in the present study.

The underlying mechanisms that render adipocytes susceptible to metabolic challenges and provoke immune responses in the adipose tissue remain largely elusive. Zonal (regional) distribution of metabolically challenged adipocytes and associated immune responses in a single fat depot could serve as a useful model for studying the underlying mechanisms. Furthermore, an understanding of the mechanisms involved in the downregulation of immune cell activity in inflamed adipose tissue in obesity might help identify molecular targets of therapeutic relevance.

## Abbreviations

CLS: Crown-like structures; HFD: High-fat diet; HOMA-IR: Homeostatic model assessment-insulin resistance; IL-6: Interleukin-6; IL-10: Interleukin-10; LFD: Low-fat diet; MCP-1: Monocyte chemotactive protein-1; mMCP6: Mouse mast cell protease-6; TNF-α: Tumor necrosis factor-α.

## Competing interests

The authors declare that they have no competing interests.

## Authors' contributions

MMA, MAR, BN, LMO and AJM researched data and reviewed/edited manuscript. KBJ and GM contributed to discussion and reviewed/edited manuscript. PZ researched data. AP and JR reviewed/edited manuscript. AN designed experiments, researched data, and wrote manuscript.
